# Bioactive Cell-Derived ECM Scaffold Forms a Unique Cellular Microenvironment for Lung Tissue Engineering

**DOI:** 10.3390/biomedicines10081791

**Published:** 2022-07-26

**Authors:** Ali Doryab, Otmar Schmid

**Affiliations:** Institute of Lung Health and Immunity (LHI) and Comprehensive Pneumology Center (CPC), Helmholtz Munich, Member of the German Center for Lung Research (DZL), 85764 Munich, Germany; otmar.schmid@helmholtz-muenchen.de

**Keywords:** lung tissue engineering, lung transplantation, decellularization, extracellular matrix, barrier integrity

## Abstract

Chronic lung diseases are one of the leading causes of death worldwide. Lung transplantation is currently the only causal therapeutic for lung diseases, which is restricted to end-stage disease and limited by low access to donor lungs. Lung tissue engineering (LTE) is a promising approach to regenerating a replacement for at least a part of the damaged lung tissue. Currently, lung regeneration is limited to a simplified local level (e.g., alveolar–capillary barrier) due to the sophisticated and complex structure and physiology of the lung. Here, we introduce an extracellular matrix (ECM)-integrated scaffold using a cellularization–decellularization–recellularization technique. This ECM-integrated scaffold was developed on our artificial co-polymeric BETA (biphasic elastic thin for air–liquid interface cell culture conditions) scaffold, which were initially populated with human lung fibroblasts (IMR90 cell line), as the main generator of ECM proteins. Due to the interconnected porous structure of the thin (<5 µm) BETA scaffold, the cells can grow on and infiltrate into the scaffold and deposit their own ECM. After a mild decellularization procedure, the ECM proteins remained on the scaffold, which now closely mimicked the cellular microenvironment of pulmonary cells more realistically than the plain artificial scaffolds. We assessed several decellularization methods and found that 20 mM NH_4_OH and 0.1% Triton X100 with subsequent DNase treatment completely removed the fibroblasts (from the first cellularization) and maintains collagen I and IV as the key ECM proteins on the scaffold. We also showed the repopulation of the primary fibroblast from human (without chronic lung disease (non-CLD) donors) and human bronchial epithelial (16HBE14o^−^) cells on the ECM-integrated BETA scaffold. With this technique, we developed a biomimetic scaffold that can mimic both the physico-mechanical properties and the native microenvironment of the lung ECM. The results indicate the potential of the presented bioactive scaffold for LTE application.

## 1. Introduction

Chronic respiratory diseases are one of the leading causes of death worldwide, for which symptom-relieving but no causal therapies exist [1]. This not only leads to a high burden of disease in the population, but also implies significant socio-economic costs [2]. Currently, lung transplantation is the only available causal therapy for chronic lung diseases, which is only available for end-stage disease [3]. The lungs are highly sophisticated to fully regenerate in the lab due to both their intricate architecture and complex function [4]. In the last decades, several strategies have been pursued to engineer a section of the lung tissue such as the alveolar–capillary barrier [5,6,7,8].

Artificial polymeric scaffolds based on synthetic and natural materials have been fabricated to imitate the extracellular matrix (ECM) in the lungs [7]. The scaffolds play a crucial role in tissue engineering (TE) development since they could be tailored to provide structural and physicomechanical support and emulate the cellular microenvironments to moderate cellular physiology [9,10]. Synthetic-based scaffolds such as poly(ε-)caprolactone (PCL) and polyethylene terephthalate (PET) have been manufactured to culture pulmonary epithelial cells [11,12,13,14,15]. On the other hand, natural-based scaffolds such as collagen (or its surrogate gelatin) and decellularized ECM (dECM) have shown excellent bioactivity and biocompatibility [7,16,17]. Nevertheless, the scaffolds based on synthetic and natural polymers suffer from a lack of cell affinity and poor physicomechanical properties, respectively [18,19]. Several studies have reported a composite of synthetic and natural polymers or hybrid scaffolds for lung tissue engineering (LTE) applications with a combination of biocompatibility and mechanical properties. [14,18,20]. However, the hybrid scaffolds failed to mimic the tissue-specific microenvironments.

We previously introduced the co-polymeric BETA (biphasic elastic thin for air–liquid culture conditions) membrane consisting of a synthetic (PCL) and natural (gelatin) polymer with an optimum mixing ratio of 9.35% PCL and 6.34% gelatin [*w*/*v* solvent] for lung application [21,22]. The BETA membrane contains a sacrificial component (gelatin), which provides enough space for the cells to deposit their own ECM during proliferation. In the present study, we exploit this feature of the BETA membrane to improve the biomimicry of the BETA scaffold. We introduce a hybrid bioactive scaffold consisting of a synthetic co-polymeric BETA membrane acting as a backbone of the ECM in the alveolar–capillary tissue, which is then biologically enhanced by cell-derived ECM to closely mimic the microenvironment of the pulmonary cells. The results show that the integrated cell-derived ECM with a supporting biomimetic membrane can not only mimic the composition and 3D structure of the lung ECM, but also enhances the physiologic biomechanical and biophysical properties of the scaffold for LTE application.

## 2. Materials and Methods

### 2.1. BETA Scaffold

The BETA scaffold was manufactured, as previously described [21]. Briefly, the copolymer emulsion of poly(ε-caprolactone) (PCL: Sigma-Aldrich, Saint Louis, MO, USA, Mn 80,000), and gelatin (Type A from porcine skin, Sigma) dissolved in TFE ((2,2,2-trifluoroethanol; Roth) were spin-coated (2000 rpm) and were dried under vacuum (300 mbar). The optimum mixing ratio of 9.35% PCL and 6.34% gelatin [*w*/*v* solvent] was used for the co-polymer of PCL/gelatin as previously described [22]. Membranes were sterilized before cell culture experiments with PBS, ethanol 80%, and UV exposure. Scanning electron microscopy (SEM, Zeiss Crossbeam 340, Carl Zeiss AG, Oberkochen, Germany) was used to study the morphology of the BETA scaffolds. The cells were fixed in 2% *v*/*v* glutaraldehyde (GA; Sigma-Aldrich) and then dehydrated in gradient acetone solutions (Sigma-Aldrich) followed by hexamethyldisilazane (HDMS, Sigma-Aldrich) for 15 min. The samples were subsequently sputter-coated with platinum and analyzed at an operating voltage of 2 kV.

### 2.2. Chick Chorioallantoic Membrane (CAM) Assay

The ex ovo chick chorioallantoic membrane (CAM) assay was employed to examine the biocompatibility and formation of ECM on the BETA scaffold. Fertilized White Leghorn chicken eggs were purchased from LSL Rhein-Main (Dieburg, Germany). After sterilization of the eggs with 80% EtOH, they were incubated at 37 °C with 60% humidity. The BETA scaffolds were placed on top of the CAM on day 6, and the eggs were resealed with parafilm. The scaffold and the surrounding tissues were excised together on day 10 and fixed with 4% PFA for 1 h at room temperature. The fixed samples were then dehydrated in a 30% *w*/*v* (overnight at 4 °C) and 15% *w*/*v* (overnight at 4 °C) sucrose solution, and embedded in an optimal cutting temperature compound (OCT Embedding Matrix, Carl Roth, Germany) for sectioning. To evaluate the ECM formation and connective tissue, Masson’s trichrome (Carl Roth, Germany) was used according to the standard protocols, and images were acquired using a Zeiss Axio Scan Z.1 slide scanner.

### 2.3. Cell Sources and Maintenance

The human epithelial type II-like A549 cell line (CCL-185) (ATCC, Manassas, VA, USA) was maintained in Dulbecco’s modified Eagle medium: Nutrient Mixture F-12 (DMEM/F12, 1:1 *v*/*v*, 2.5 mM L-glutamine, 15 mM HEPES, Gibco, Thermo Fisher Scientific, Waltham, MA, USA) supplemented with 10% FBS (Gibco) and 1% (*v*/*v*) Pen/Strep (100 U mL^−1^, Gibco). The IMR-90 human lung fibroblast cell line (ATCC) was cultured and maintained in DMEM/F12 supplemented with 10% FBS and 1% (*v*/*v*) Pen/Strep. The human bronchial epithelial 16HBE14o^−^ cell line from cystic fibrosis (SCC150, Millipore, Sigma-Aldrich, Temecula, CA, USA) was cultured and maintained in minimum essential media (MEM, Gibco) supplemented with 10% FBS and 1% *v*/*v* Pen/Strep (100 U mL^−1^). The primary human lung fibroblasts (phLFs) were derived from n = 3 patients without chronic lung disease (non-CLD) that underwent surgery at the Thoracic Surgery, LMU Hospital and the Asklepios Pulmonary Hospital Munich-Gauting. The study was approved by the local ethics committee (LMU, CPC-M bioArchive, 19-630) and written informed consent was obtained from all patients. The phLFs were isolated from lung explants or tumor-free areas of the lung resections as previously described [23]. The phLFs were cultured and maintained in DMEM/F12 supplemented with 10% FBS and 1% (*v*/*v*) Pen/Strep. Mouse embryonic fibroblasts (MEF) were maintained in DMEM/F12 high glucose supplemented with 10% FBS and 1% (*v*/*v*) Pen/Strep.

### 2.4. ECM-Integrated BETA Scaffold

IMR90 cells with a cell density of 40,000 cells cm^−2^ were seeded on a BETA scaffold and grown in a culture medium of DMEM/F12 supplemented with 10% FBS, 1% L-glutamine (2 × 10^−3^ M, Gibco), 2-phospho-L-ascorbic acid (0.1 × 10^−3^ M, Sigma), and 1% (*v*/*v*) Pen/Strep. The cells were also treated with 5 ng mL^−1^ recombinant human TGFβ1 protein (R&D Systems, Minneapolis, MN, USA) to enhance ECM production. After 5 days, the cells were decellularized at 37 °C, as described in Table 1, based on the literature [24,25,26,27]. After decellularization, the BETA scaffolds were washed three times with PBS. To prevent unwanted DNA, the acellular BETA scaffolds were incubated with 10 U/mL DNAse I, RNase-free (Thermo Scientific EN0525) in PBS with Ca^2+^, and Mg^2+^ for 1 h at 37 °C. After decellularization and DNAse treatment, the ECM-integrated BETA scaffolds were used for the repopulation step. Among the decellularization protocols, the optimized method (*20 mM NH_4_OH and 0.1% Triton X-100*), was performed and the ECM-integrated BETA scaffold was subsequently repopulated with the phLFs with a cell density of 40,000 cells cm^−2^ in the culture medium of DMEM/F12 supplemented with 10% FBS and 1% (*v*/*v*) Pen/Strep for 5 days. The scaffolds were also seeded with the human bronchial epithelial 16HBE14o^−^ cell line with cell density and 150,000 cells cm^−2^ in the culture medium of MEM (Gibco) supplemented with 10% FBS and 1% *v*/*v* Pen/Strep (100 U mL^−1^) for 6 days. The samples were then fixed with 4% paraformaldehyde (PFA; Sigma-Aldrich) for 20 min at room temperature for IF analysis.

The WST1 colorimetric metabolic activity assay (Roche, Mannheim, Germany) was used to evaluate the cell viability. Briefly, 1 mL of diluted WST1 reagent (1:10) was incubated with the cells for 20 min and the supernatant (200 µL) was transferred to a 96-well plate. The absorbance was measured at 450 nm (Safire2TM, Tecan, Männedorf, Switzerland).

### 2.5. Functional Analysis

The transepithelial electrical resistance (TEER) measurement (Millicell ERS-2, Millipore, Burlington, MA, USA) was used to assess the barrier integrity in 16HBE14o^−^ cells. To obtain TEER values (Ω cm^2^), the measured resistance in ohm (Ω) is subtracted from the resistance of the membrane (without cells) and then multiplied by the effective growth surface area (cm^2^; here 4.2 cm^2^).

To measure the apparent permeability, FITC-dextran 4 kDa (FD4, Sigma-Aldrich) (1 mL in PBS) was added to the apical side of the membrane (surface area 4.2 cm^2^). Every 30 min, 100 µL of FD4 in the basal side (total volume 2 mL) was taken in a black 96-well plate and analyzed by the plate reader (Safire2^TM^, Tecan; excitation: 483 nm, emission: 525 nm). The apparent permeability coefficient (*P_app_*, cm s^−1^) was calculated with Equation (1).

(1) $$P_{app}=\frac{\mathrm{dQ}}{\mathrm{dt}}\times \frac{1}{\left(A*C_{0}\right)}$$

where dQ/dt is the steady-state flux or the transport rate;

*A* is the surface area of the membrane;

*C*_0_ is the initial concentration of FITC-dextran 4 kDa (FD4, Sigma-Aldrich) added to the apical compartment (mg mL^−1^).

### 2.6. Immunofluorescence (IF)

For the immunofluorescence (IF) analysis, the cells on the membrane, the decellularized membrane, and the repopulated membranes were fixed with 4% PFA, washed with PBS, and permeabilized by 0.3% Triton X-100 (Sigma-Aldrich) in PBS. The samples were then incubated with primary antibodies overnight at 4 °C and subsequently with secondary antibodies in a blocking buffer. The primary antibodies were anti-alpha smooth muscle actin (αSMA) Ab (1:250, mouse, Abcam, Cambridge, UK, cat. # ab7817), Ki67 monoclonal (1:100, mouse, eBioscience, Invitrogen, Waltham, MA, USA, cat. # 14-5698-82), collagen type I (1:100, rabbit, Rockland, cat. # 600-401-103-0.1), and collagen type IV monoclonal (1:100, mouse, eBioscience, Invitrogen, cat. # 14-9871-82). The secondary antibodies were Alexa Fluor 488 goat anti-mouse (Invitrogen, cat. # A-11001, 1:250) and Alexa Fluor 568 goat anti-rabbit Ab (Invitrogen, cat. # A-11011, 1:250). The F-actin cytoskeletons were stained with Alexa Fluor 568 Phalloidin (1:400, Invitrogen, cat. # A12380) and Alexa Fluor 488 Phalloidin (1:400, Invitrogen, cat. # A12379). The cell nuclei were stained with 4′,6-diamidino-2-phenylindole (DAPI, 1:250, Sigma-Aldrich, cat. # D9542). The samples were then embedded in Glycergel (DAKO Schweiz AG, Baar, Switzerland). All cell images were acquired using Zeiss 880 upright confocal laser scanning microscopy (CLSM) coupled with ZEN Black 2.3. Images were further processed using the 3D reconstruction ImageJ/Fiji, v 2.3.0/1.53f, National Institutes of Health (NIH), Bethesda, MD, USA.

## 3. Results

**Figure 1:**
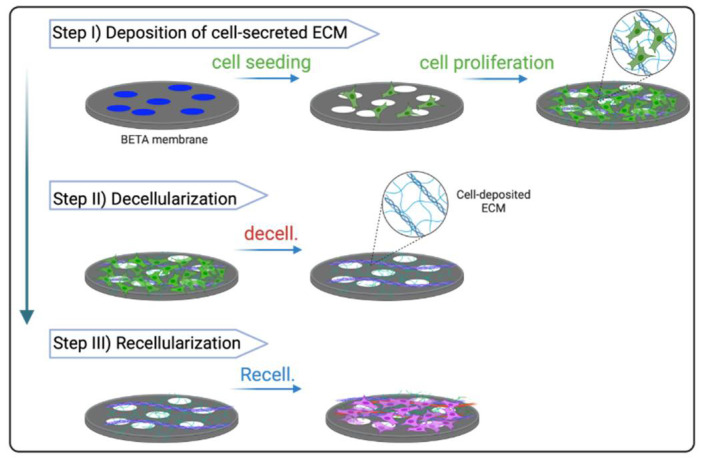
The concept of the ECM-integrated BETA scaffold. First, the biphasic elastic thin for air–liquid culture conditions (BETA) scaffold is populated with the human lung fibroblast cell line (IMR90). After the formation of a confluent cell layer, the membrane is gently decellularized to maintain the aligned and linear proteins of the cell-secreted ECM such as collagens while removing the DNA materials of the fibroblasts. Finally, the hybrid construct of the BETA scaffold and cell-secreted ECM or ECM-integrated BETA scaffold is recellularized with the target pulmonary cells. The schematics were created with BioRender.com (accessed on 27 June 2022).

**Figure 2:**
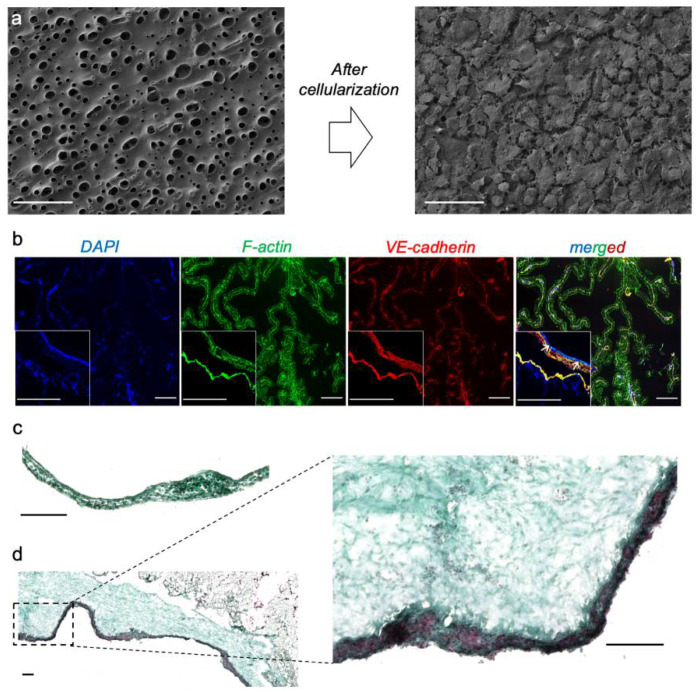
The biocompatibility of the BETA scaffold. (**a**) Scanning electron microscopy (SEM) of the BETA scaffold, which has already been introduced by us elsewhere [21]. The cells were populated onto and into the porous BETA scaffold with alveolar epithelial type II-like A549 cells within 5 days. The scale bars of the SEM images are 50 µm. (**b**) The chick chorioallantoic membrane (CAM) assay was used to evaluate the biocompatibility of the BETA scaffold. The immunofluorescence (IF) analysis showed the formation of blood vessels on the BETA scaffold characterized by VE-cadherin (red). Cell nuclei are shown in blue (DAPI) and F-actin cytoskeleton in green. (**c**,**d**) The CAM-deposited ECM and connective tissue on the membrane analyzed by Masson’s trichrome analysis. (**c**,**d**) show the cross-sectioned and diagonally cross-sectioned view of the CAM and BETA scaffold, respectively. Cell nuclei are shown in dark blue/dark brown and connective tissue in green. The scale bars in (**b**–**d**) are 100 µm.

Here, we developed a bioactive scaffold based on cell-derived ECM, which was integrated with the BETA membrane (Figure 1). The BETA membrane has already been introduced by us, which is thin (thickness is 5 µm) with high wettability (WCA: 68 ± 5) [21]. The BETA membrane is stretchable with an elastic modulus of 0.78 ± 0.24 MPa and its fatigue test showed no plastic deformation or creep [21]. The BETA has 10% surface porosity and permeability of 8.18 ± 2.54 × 10^−6^ cm s^−1^ for 4 kDa FITC-dextran [21]. The cytocompatibility of the BETA membrane has been evaluated using lung epithelial cell lines such as human lung alveolar epithelial (A549) and human bronchial epithelial (16HBE14o^−^) cells, which showed no systematic toxicity or leaching of unwanted materials to the culture medium (Figure 2a) [21,22].

**Figure 3:**
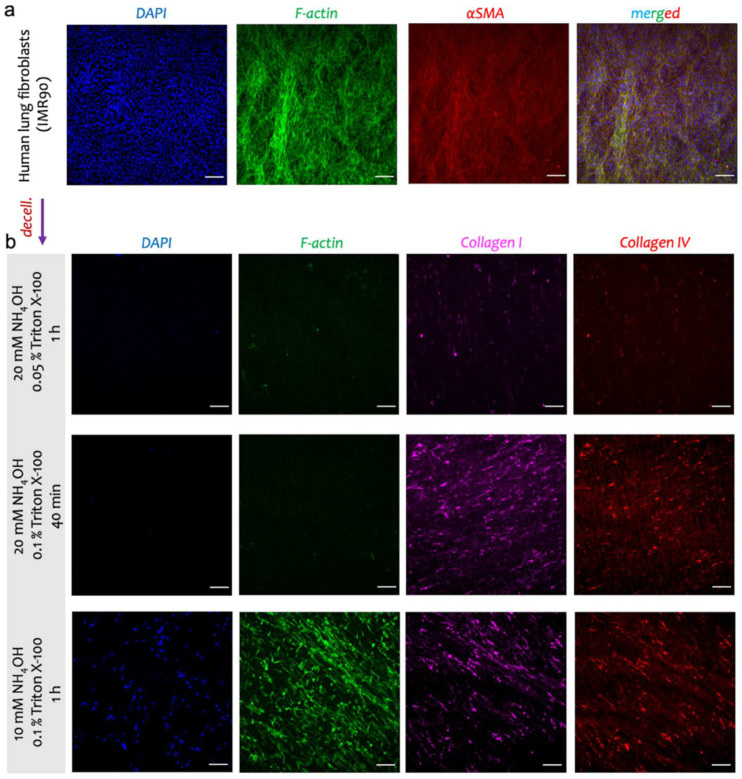
The IF analysis of the ECM-integrated BETA membrane. (**a**) the IMR90 human lung fibroblast cells were cultured on the BETA membrane and treated with 5 ng mL^−1^ TGFβ1. The activated fibroblasts (myofibroblasts) characterized by αSMA (in red) produced high amounts of ECM proteins in response to a fibrotic agent of TGFβ1. (**b**) The populated scaffolds with IMR90 cells were subsequently decellularized. The selected acellular BETA membrane shows the complete or partial removal of cells retaining collagen I and IV as the two key ECM proteins. Protocol 2 (*20 mM NH_4_OH and 0.1% Triton X-100* for 40 min) with DNAse I treatment (100 U/mL in PBS with Ca^2+^ and Mg^2+^) was preferred as the optimized decellularization protocol. Details of the decellularization protocols are shown in Table 1. Collagen I (magenta), collagen IV (red), F-actin cytoskeleton (green), and cell nuclei (DAPI, blue). Scale bars are 100 µm.

**Figure 4:**
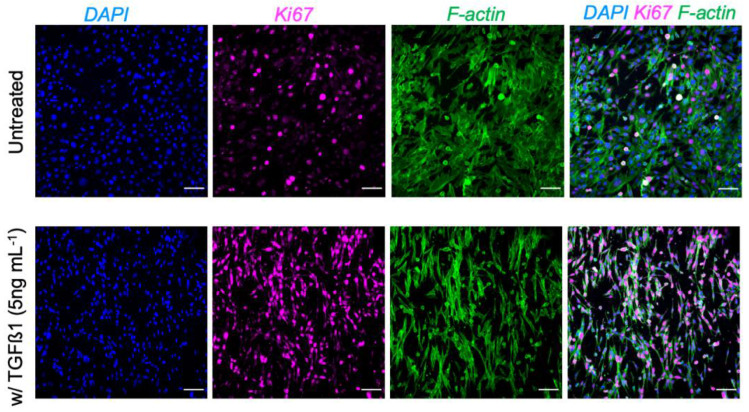
The IF analysis of mouse embryonic fibroblasts (MEF) on the BETA membrane without and with the treatment with TGFβ1 as a fibrotic agent. *Ki67*, which is associated with cellular proliferation, is amplified after the induction of MEF with 5 ng mL^−1^ TGFβ1. Ki67 (magenta), F-actin cytoskeleton (green), nuclei (DAPI, blue). Scale bars are 100 µm.

In the present study, we also showed the biocompatibility of the BETA using the chick chorioallantoic membrane (CAM) assay (Figure 2b–d). The analysis of the surrounding tissues/BETA showed the deposition of connective tissue and ECM proteins (such as collagen) on the BETA scaffold (Figure 2c,d). According to the CAM assay, we also found that the cells could deposit their own ECM onto the BETA scaffold (Figure 2c,d). Masson’s trichrome analysis showed that fibrous connective tissues are resigning on and within the BETA membrane. To obtain a bioactive scaffold, we first populated the BETA membrane with the IMR90 human pulmonary fibroblast cell lines to deposit the ECM proteins into the scaffold (Figure 3a). It has been shown that transforming growth factor-β1 (TGF-β1) stimulates pulmonary cells to produce matrix proteins [26]. Thus, we stimulated the fibroblasts with TGFβ1 as a fibrotic agent to stimulate the innate production of their own ECM proteins into the BETA membrane. We showed that the treatment of mouse embryonic fibroblasts (MEF) on the BETA membrane with 5 ng mL^−1^ TGFβ1 upregulates cell proliferation and modulates the fibroblast phenotype, as indicated by Ki67, which is associated with cellular proliferation (Figure 4).

After almost 3 days of cell culture, the gelatin was completely dissolved from the BETA membrane (PCL/gelatin), leaving pores for the expansion of cells and their ECM (Figure 2a, left panel). The IF analysis showed the formation of a confluent layer of activated fibroblasts on the BETA scaffold, characterized by the F-actin cytoskeleton and αSMA (a marker of myofibroblast) (Figure 3a). The populated membrane with fibroblasts were subsequently (gently) decellularized to preserve the key proteins of ECM and improve the bioactivity of the BETA scaffold (Figure 3b). We followed several protocols for decellularization by changing the detergent type, the concentration of detergent, and the decellularization time, which are widely used in the literature [24,25,26,27]. Both *NH_4_OH* and *Triton X-100* are typically considered as mild decellularization agents compared to the invasive sodium dodecyl sulfate (SDS) [24,25,26,28]. The full details of the protocols are provided in Table 1.

**Figure 5:**
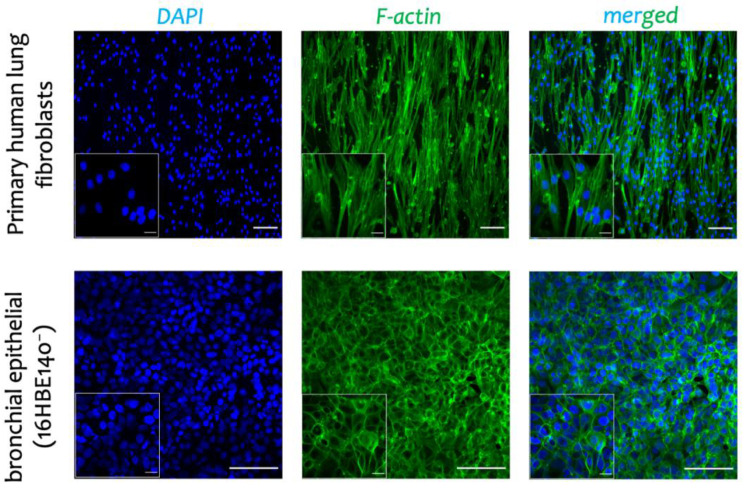
The recellularization of the ECM-integrated BETA scaffold with pulmonary cells. The ECM-integrated BETA scaffolds were repopulated with (**top**) the primary fibroblasts from human (non-CLD donors) and (**bottom**) human bronchial epithelial (16HBE14o^−^) cells on the ECM-integrated BETA scaffold. F-actin cytoskeleton (green) and cell nuclei (DAPI, blue). Scale bars in the large and small panels are 20 and 100 µm, respectively.

The IF analysis of the three main protocols showed that protocol 2 (*20 mM NH_4_OH and 0.1% Triton X-100 for 40 min)* is a superior protocol in terms of the complete removal of the cells (both cell nuclei, DAPI, and F-actin cytoskeleton) and at the same time, preserving collagen I and IV, the main proteins of the ECM (Figure 3b). To fully remove the DNA materials, which harms the further recellularization step, we incubated the acellular scaffold with DNase I for 1 h. We used this *optimized* protocol for the recellularization step. To assess the feasibility of the re-population of cells on the decellularized scaffold, we used primary fibroblasts from human (without chronic lung disease (non-CLD) donors) and human bronchial epithelial (16HBE14o^−^) cells. The re-populated scaffold with primary fibroblasts derived from non-CLD patients showed the formation of the F-actin cytoskeleton (Figure 5 top panel) with almost no cytotoxicity effect (WST-1 assay, data not shown). The IF analysis showed the formation of a monolayer of 16HBE14o^−^ as characterized by the F-actin cytoskeleton (Figure 5b bottom panel). The transepithelial electrical resistance (TEER) and apparent permeability of the monolayer of 16HBE14o^−^ were measured as 827 ± 178 Ω cm^2^ and 4.2 × 10^−6^ cm/s, respectively.

## 4. Discussion

Several bottom–up and top–down approaches have been used to engineer lung tissue. Whole or partial organ decellularization is a highly promising method to maintain the main structure and key proteins of the lung, which then facilitates the recellularization of autologous stem or progenitor cells [8,29,30,31,32]. The application of whole organ decellularization, however, has been limited due to the immunogenicity of the decellularized scaffold during the recellularization process [33,34]. In addition, one of the main challenges of the available approaches toward functional lung tissue is the inadequate alveolar−capillary barrier function in the constructed tissue due to the lack of biomimetic scaffold [29,35].

Here, we introduced a hybrid bioactive scaffold to closely mimic the microenvironment of the alveolar–capillary barrier. The hybrid bioactive scaffold consisted of (i) BETA scaffold and (ii) ECM-derived cells. In the co-polymeric BETA scaffold of PCL/gelatin, we used PCL, which is widely used in TE and regenerative medicine applications, mainly due to its biocompatibility, mechanical properties, and suitability for modification. The application of PCL for the pulmonary cells has been shown by us [21,22] and other groups [11,14]. In the BETA scaffold, we also adopted gelatin as a delayed dissoluble porogen to (i) promote initial cell attachment by integrin-mediated interactions via its cell-responsive motifs such as arginine–glycine–aspartic acid (RGD) sequence, and (ii) provide a space for cell infiltration after (gelatin) removal, leaving a cavity. Gelatin is a bioresorbable and reproducible polymer with tunable biophysical properties and very low immunogenicity compared to native ECM polymers such as collagen [16,36]. The PCL/gelatin membrane has been designed to recapitulate the main physico-mechanical characteristics of the alveolar–capillary barrier—the gas exchange area in the lung. The BETA scaffold is also self-supported in the absence of any toxic cross-linker.

We have already shown that BETA has connective porosity by using focused ion beam-scanning electron microscopy (FIB-SEM) tomography, which allows the cells to infiltrate and reside in the scaffold, interact with other cells, and form their own ECM materials [21]. We exploited this advantage of the BETA membrane to make it even more biomimetic through the deposition of the natural ECM proteins by pulmonary cells (Figure 1). The deposition of cell-derived ECM onto synthetic scaffolds is a promising approach to mimicking the microenvironment and natural complexity of the region of interest with favorable biological characteristics [37]. Among the human lung fibroblasts, IMR90 (a fibroblast isolated from the normal lung tissue) has shown a great potential to produce a high density of cell-derived ECM [27]. We therefore employed IMR90 as a sacrificial ECM generator for the biomimetic scaffold. We stimulated the fibroblasts with a profibrotic agent of TGFβ1 to differentiate them from myofibroblasts (characterized by αSMA marker, see Figure 3a). The activated fibroblasts (myofibroblasts) can produce high amounts of ECM proteins in response to pro-fibrotic stimuli [38].

The analysis of the barrier integrity dynamics, measured by TEER and apparent permeability, showed that the monolayer of 16HBE14o^−^ cells on the bioactive BETA scaffold could form a tighter barrier compared to the established models on Transwell inserts. The TEER value obtained for 16HBE14o^−^ was almost 2-times higher than the already reported data in the literature [39,40]. The apparat permeability also showed a firmer paracellular barrier against FITC-dextran (4 kDa), which is in line with the reported value in the literature with a range of 1.5–4 × 10^−6^ cm/s [39,40].

The collagens (types I, III, and IV) and elastin, fibronectin, and glycosaminoglycans (GAGs) are the main components of the ECM, which play a key role in the proliferation, differentiation, and migration of pulmonary cells during physiological and non-physiological (disease) conditions [38]. The deposited ECM scaffold showed an excellent regeneration of the linear ECM proteins (such as collagen I), indicating the high degree of physiologic relevance and ECM microenvironment in the lung region, in particular, the linear pattern of the ECM during disease, which is not obtained by solubilized ECM such as Matrigel or hydrogels [27]. The ECM-derived scaffolds shown enhanced cell infiltration and favorable immune responses for the cell hemostasis upon in vivo implantation [41].

Here, we developed a bioactive scaffold based on the ECM-derived human cells. It has been shown that human-based cell models can improve the predictivity of the in vitro models for drug development [42]. This feature is one of the main advantages of our ECM-derived scaffold over the already developed ones based on the decellularized animal models [8,31]. Using this method, the unique niches of the region of interest can be explored, for example, a specific disease can be mimicked due to the formation of the native ECM proteins, which can subsequently guide the cells during proliferation, migration, and infiltration. The supporting BETA membrane mimics the pore size and elastic modulus of the basement membrane in the alveolar-capillary region [21], which enhances the functionality of the regenerated tissue. This is very important since obtaining a connective porous structure is not easily accessible via only ECM-derived or natural-based scaffolds.

In summary, our bioactive scaffold benefits from (i) the biomimetic microenvironments for the cells due to the cell-deposited ECM, and (ii) an interconnected porosity for migration and special reorganization of the cells, thanks to the supporting BETA membrane. The bioactive BETA scaffold presented excellent bioactivity for pulmonary epithelial cells (16HBE14o^−^) and primary fibroblasts that were derived from non-CLD patients. The pulmonary epithelial cells could also form a tight barrier integrity, which is one of the main challenges in engineered lung tissue.

## 5. Outlook

The BETA membrane has already been shown its ability for particokinetic and pharmacokinetic studies in the lung [22]. In the present study, we extended its potential application for LTE as an allograft or xenograft transplantation by improving its biomimicry. We used a human fibroblast cell line to deposit ECM onto a synthetic BETA membrane. However, fibroblasts derived from patients with idiopathic pulmonary fibrosis (IPF) can also be employed to mimic the pathological pattern of the ECM during disease conditions such as fibrosis with higher stiffness. Moreover, the introduced cell-derived ECM scaffold is tunable and can be adjusted for other specific-tissue applications.

## Figures and Tables

**Table 1:** The decellularization protocols used in this study.

Protocol #	Description		
	Decellularization	Duration	Post-Treatment
**1**	20 mM NH_4_OH, 0.05% Triton X-100	1 h	100 U/mL DNAse I in PBS with Ca^2+^ and Mg^2+^ for 1 h
**2**	20 mM NH_4_OH, 0.1% Triton X-100	40 min	
**3**	10 mM NH_4_OH, 0.1% Triton X-100	1 h	
**4**	1 mM CHAPS and 0.1% Triton X-100	1 h	
**5**	0.1% SDS and 0.1% Triton X-100	1 h	

## Data Availability

All data supporting the findings of this study are available within the article or from the corresponding author upon reasonable request.
